# Recording, Storage, and Reproduction of Information on Polyvinyl Chloride Films Using Shape Memory Effects

**DOI:** 10.3390/polym13111802

**Published:** 2021-05-30

**Authors:** Alexander P. Kondratov, Egor P. Cherkasov, Vladislav Paley, Alex A. Volinsky

**Affiliations:** 1 Department of Innovative Materials in the Print Media Industry, Moscow Polytechnic University, ul. Bolshaya Semenovskaya, 38, 107023 Moscow, Russia; egr1987@rambler.ru; 2 Department of Mechanical Engineering, University of South Florida, 4202 E. Fowler Ave. ENG 030, Tampa, FL 33620, USA; paleyv@usf.edu

**Keywords:** shape memory polymer, internal stresses, recording and storage of information, screen printing machine, shrink film of polyvinyl chloride, tetrahydrofuran, absorption

## Abstract

Partial dissolution and plasticization are used for recording, storing, and reproducing information with modified industrial screen-printing equipment and aqueous solutions of colorless organic liquids on small surface area shape memory polymer films. To justify the choice of “ink” and evaluate the effectiveness of using organic liquids as high-speed polymer solvents, the new method for recording hidden information uses the calculation of the solubility parameter, differential scanning calorimetry, and the method of one-sided swelling of films under isometric conditions. Using the example of tactile marking of shrink labels made of polyvinyl chloride, the optimal conditions for recording hidden information on a film are established in terms of the concentration of an aqueous solution of tetrahydrofuran, the contact time, and the processing temperature of the polymer using screen printing equipment.

## 1. Introduction

Shape memory materials (SMMs) have found many applications in a wide array of industries including medicine, electrical engineering, space, aviation, textile, and automotive. These materials have an “adjustable” property that can be controlled when a specific stimulus is applied. These SMMs can be found in some everyday common devices and locations. In medicine, shape memory polymer (SMP) films are used as membranes for dosage forms [1,2], drug delivery systems [3,4,5], smart sutures and stents [6], in multiple cardiovascular applications [7,8], for antibacterial functions [9], and minimally invasive surgeries [10]. In aerospace [11,12,13], SMMs are used as active load-bearing elements in deployable panels [14], release devices during rocket launches [15], noise-reducing jet nozzles [16], aerodynamic load suppression systems [16,17], bilinear controllers [18], phase-changing light-responsive components [19], and various actuators [20,21,22]. In the automotive industry [23,24], SMMs can adjust mirrors [25], are used in battery cells [26], in electric motors [27], and in rotary engines [28]. In manufacturing warehouses, SMPs and SMP films can be found as printing labels, large-scale packaging, and individual packaging [29,30,31,32].

The need for tactile labeling of household goods such as disinfectants, cosmetics, paints, prepackaged food, assembly parts and hand tools, is evident [29,30,31,32]. Tactile marking allows ordinary consumers and people with disabilities, including the blind or visually impaired, to identify items and verify the authenticity of consumer products. Touch identification through tactile markings simplifies the management of cables and pipelines located in hard-to-reach and dimly lit areas. The formation of embossed symbols on objects and materials is possible in various ways using existing technology [33,34]. Reliefs on heat-resistant sheets and roll paper materials can be formed using printing equipment for gravure and screen printing, and now 3D printers [35,36,37,38,39,40,41,42].

Printing and creating relief on fusible and heat-shrinkable materials, which are widely used for manufacturing product labels and casings, by traditional methods, is impossible due to their low resistance to heat. For marking products made of heat-shrinkable materials, an original method of local isometric heat treatment has been proposed, which allows the use of paints and additional consumables to form relief images and Braille characters [29,35]. Tactile (embossed) marking of heat-shrinkable materials is based on two main operations. The first is local heat treatment under isometric conditions at a certain temperature and exposure time under pressure. The second is complete heating of the entire label or package at a temperature exceeding the glass transition temperature of the polymer [43].

The geometric parameters of tactile marking on heat-shrinkable materials are determined by the magnitude and anisotropy of the deformation in terms of the reduction in size (shrinkage) of the material during processing with automated applicators utilizing a stream of hot air or superheated water vapor [44]. Thermally stimulated deformation of a heat-shrinkable material depends on the supramolecular structure of the polymer and the rate of relaxation of internal stress in heat-shrinkable films subjected to local isometric heat treatment at the marking stages [35].

It is well known that the relaxation rate of films upon heating depends on the ambient temperature and their composition [45]. The hypothesis put forward in this paper is that it is possible to accelerate the relaxation process in local areas of heat-shrinkable films, which form a relief when heated, by short-term treatment using a liquid of high thermodynamic affinity with the polymer, i.e., a good volatile solvent or plasticizer [46,47]. In the original research papers devoted to changing the shape of polymeric materials upon heating or as a result of plasticization with solvents, the temperature of materials and the shape of samples with shape memory change when the sample is completely immersed in the coolant or solvent in a free state, with no force or dimensional constraints [48,49,50].

The qualitative difference between the processes underlying the recording of information on materials with shape memory presented in this paper is that both mechanisms for starting the process of restoring the shape of films with shape memory are carried out locally sequentially and are combined in a special way. A special procedure consists of applying a liquid to a film using a screen printing method. The screen printing technology is easily implemented and is suitable for large-scale production [51]. Screen printing technology has already been used to record relief information on porous sheet materials [52]. It potentially allows for inexpensive Braille printing on regular office paper that would otherwise use expensive specialty paper. At the same time, the effects of paper porosity and spacing between the mesh threads in a stencil printing form were investigated [53].

The theoretical consideration of the mechanism of liquid penetration through stencil-shaped capillaries, depending on their diameter and the structure of the surface of the threads, is of particular scientific interest. In [54], a fractal model of a capillary fluid flow in a fibrous porous medium is proposed, which allows one to take into account the role of the surface roughness of the filaments. The advantage of the physical mechanisms of capillary flow through a single convoluted capillary with a rough surface in a fibrous-porous medium is that it does not contain empirical constants, which are required in other models. The amount of liquid applied to the film through a stencil mesh depends on the mesh permeability and the viscosity of the solutions [52]. A mathematical model of the process of permeability of fibrous porous media was proposed in [55]. The model allows establishing the dependence of the permeability of a fibrous-porous medium on the fractal dimensions of the pore area. The proposed fractal model provides a better understanding of fluid transport through fibrous porous media.

The next stage in the implementation of recording relief information on films is to study the kinetics of liquid penetration into the polymer, change the structure of the film and reduce the level of internal stresses causing heat shrinkage. To create a new technology for recording information on films with shape memory, it is necessary to develop experimental methods for determining the amount of liquid that penetrates into the polymer upon contact with one side of the film. It is important to study the effects of the concentration of aqueous solutions of organic substances and the temperature of the solutions on the kinetics of liquid penetration into the film through the porous structure of the stencil mesh printing equipment.

The purpose of this paper is to experimentally test the hypothesis and justify the possibility of using commercial printing equipment for recording, storing, and reproducing information on SMP films employing short-term liquid treatment.

## 2. Materials and Methods

To obtain the initial data necessary for the development of the tactile marking method, 75 μm thick polyvinyl chloride (PVC) shrink film (PVCLF-T147/07 T25, Klockner Pentaplast, Montabaur, Germany) was used with a 120 mm flat sleeve width. The film contained copolymers of vinyl chloride and vinyl acetate with a composition of 90% vinyl chloride copolymer, 4% dioctyl phthalate, calcium stearate, 3% polyphenylmethylsiloxane, and 3% epoxidized soybean oil. Tetrahydrofuran (THF) (EKOS-1, Moscow, Russia) was used as a solvent for the copolymer. For covert marking and recording information by short-term contact with a THF solution, two methods were used.

Samples for studying recording and reproducing information on films made of polyvinyl chloride with shape memory effect were made using laboratory and industrial equipment. Laboratory samples were used to assess the supramolecular structure and internal stresses of an SMP film by differential scanning calorimetry (DSC). It is a macro model of a film region treated with a solvent with fixed dimensions in the direction of shrinkage, which forms a relief after heat treatment. A macro model of a film area wetted with a solvent and forming a relief after heat treatment is a shape memory film sample at a 100:1 scale with about 100 cm^2^ surface.

**Figure 1:**
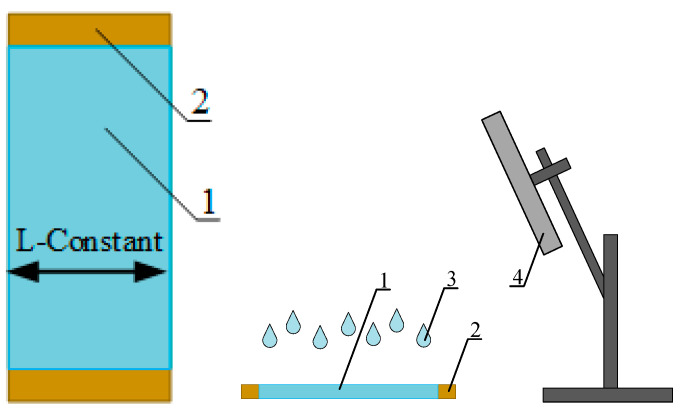
Schematics of model film samples preparation for DSC analysis. 1—film, 2—mandrel for fixing the film size along the shrinkage direction, 3—solvent, 4—air flow stimulator, 4—vacuum evacuation (not shown).

The preparation of the laboratory samples included processing in a shell film on a rigid mandrel (Figure 1) with aqueous tetrahydrofuran solution, drying, and evacuation to constant weight. The shell prevented the solvent from triggering the shape recovery mechanism.

The laboratory version of the samples is designed to assess the exothermal effect of shrinkage and to determine the enthalpy of melting. While this information is not the main purpose of the paper, it is presented to explain the essence of the phenomena of the hidden recording of information and its manifestation in the form of relief.

**Figure 2:**
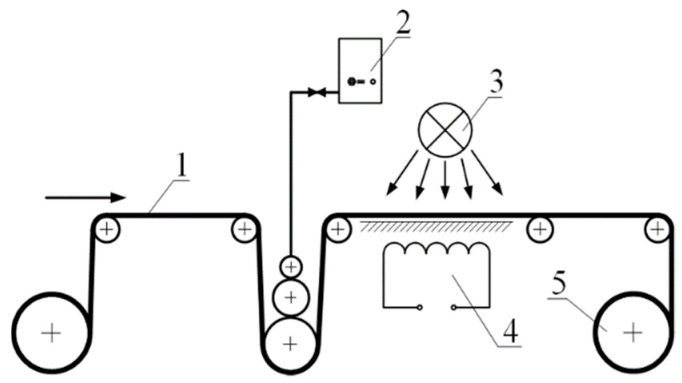
Schematics of tetrahydrofuran solution application, drying and storage in a roll. 1—film, 2—container with solution, 3—fan, 4—thermostat, 5—roll.

**Figure 3:**
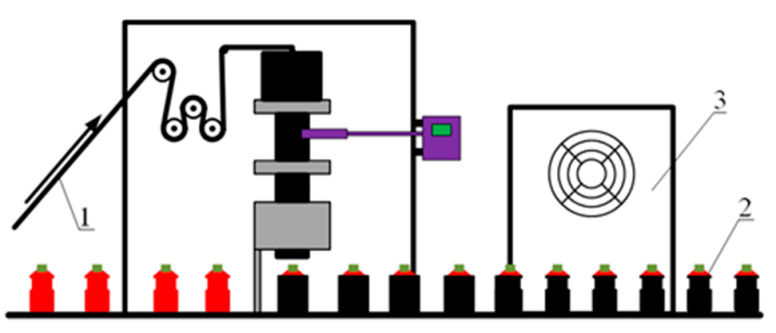
Schematics of heat shrinkage of films with hidden marking on containers using industrial equipment. 1—film with hidden marking, 2—container, 3—thermostat.

**Figure 4:**
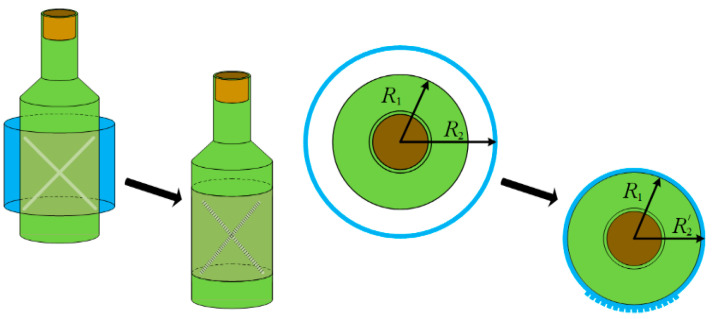
The manifestation of relief after heat shrinkage of a film with hidden markings on a cylindrical container. R_1_, R_2_, R_2′_ are dimensions of containers and shells on containers before and after heat shrinkage.

The second production version of the samples is a tape of indefinite length for local (point) application of an aqueous tetrahydrofuran solution, drying and storage in a roll (Figure 2), and subsequent shrinkage on containers using printing equipment (Figure 3 and Figure 4). The fraction of the surface area of the film wetted with a tetrahydrofuran solution does not exceed 1%. This guarantees the mode of isometric processing of the polymer with a solvent and the absence of shrinkage (appearance of relief) at the stage of hidden information recording.

After a short exposure to the plasticizer penetrating the polymer film or partially dissolving the surface layer of the polymer film with shape memory, the samples were thoroughly dried. The weight of the samples was stable and did not change during drying and evacuation. In the authors’ opinion, there is no reason to attribute the decrease in the enthalpy of melting of the films after solvent desorption to the effect of weight loss during DSC samples analysis.

The first method used a laboratory pad printing machine with a fibrous-porous printing element impregnated with a liquid, pressed against the sample for a fixed time [56]. The second method utilized a modified section of commercial printing equipment for screen printing. To reveal hidden markings, a thermostat (Binder ED 115 thermostat, Tuttlingen, Germany), an air chamber for shrinking labels [44], and a water thermostat were used.

Liquid varnishes were used here for printing on polymer films, as solvents and plasticizers compatible with PVC in the Heidelberg printing press. The composition of the varnishes included cyclohexanone, o-xylene, butanol-1, 1-methoxy-2-propanol, 1-butoxypropan-2-ol, 4-hydroxy-4-methylpentan-2-OH, toluene, ethyl acetate, butyl glycolate, 2-methoxy-1-methylethyl acetate, 2-butoxyethyl acetate, 2-methoxy-propyl acetate, and other esters and saturated hydrocarbons of various fractions. For a preliminary assessment of the applicability of the liquid components of varnishes and paints, the solubility parameters were calculated from their chemical structures and the thermodynamic compatibility of these liquids with PVC was determined [57].

By calculation and experimental evaluation of the sorption of liquids by a film, it has been established that PVC is thermodynamically compatible with cyclohexanone, THF, o-xylene, carbon tetrachloride, alcohols, and esters, which are parts of many paints and varnishes. The maximum sorption rate and solubility of PVC occur in contact with THF. To control the rate of sorption processes and prevent the dissolution of the film, a mixture of THF and distilled water was used. Solutions with various THF concentrations of 30, 40, and 50% were prepared in distilled water.

All concentrations of solvents at all temperatures have been investigated, but since the purpose of this paper is to show how printing and labeling equipment can be used for packaging goods to record information in the form of relief, only the optimal conditions and results are provided.

In order to simulate the process of short-term interaction of the THF aqueous solutions with the PVC film, a previously developed technique for accurately fixing the contact time and size of film samples (isometric mode of film swelling and/or partial dissolution) was modified [58]. A shell was made in order to comply with isometric conditions. A sleeve was welded from a heat-shrinkable film, with a 1/3 mm thick rigid mandrel placed inside. The mandrel prevented the film from shrinking when heated and exposed to liquids. The technique included placing a sample of a heat-shrinkable film in the form of a shell on a mandrel under a sealed cover. The container with the THF solution was placed in a thermostat to maintain a constant temperature and when the set temperature was reached, the container was turned upside-down (180°), allowing the solution to contact the PVC film. The contact time of the solution with the film was determined by the holding time of the container in an inverted state, ranging from 1 to 10 min. After a specified exposure time, the container was turned back over to its original position, the container lid was removed, and the film sample was immersed on the frame in distilled water which instantly removed the excess THF from the film surface. Thus, the exact contact time of the THF solution was achieved.

To determine the effects of the THF solution on the internal stresses and the structure of the film, calorimetric studies of the films were carried out using a differential scanning calorimeter (DSC, PC-DSC 204 Phoenix, NETZSCH, Germany). A series of 10 identical samples were prepared from PVC films before and after isometric treatment with an aqueous THF solution. The samples were cut out from multiple sections of the film with a stamp in the form of microdisks with a 2 mm diameter. Film samples that were 75 μm thick and weighed 10–12 mg were placed in the crucible of the calorimeter. Thermograms were recorded at a crucible heating rate of 10 °C/min. Cooling and reheating of the samples were carried out at the same rate of 10 °C/min. The average enthalpy values of the exothermic and endothermic processes were obtained. After a short exposure to the plasticizer penetrating the polymer film or partially dissolving the surface layer of the polymer film with shape memory, the samples were thoroughly dried. The weight of the samples was stable and did not change during drying and evacuation. Thus, there is no reason to attribute the decrease in the enthalpy of melting of the films after solvent desorption to the effects of weight loss due to evaporation during calorimetry analysis.

## 3. Results

**Figure 5:**
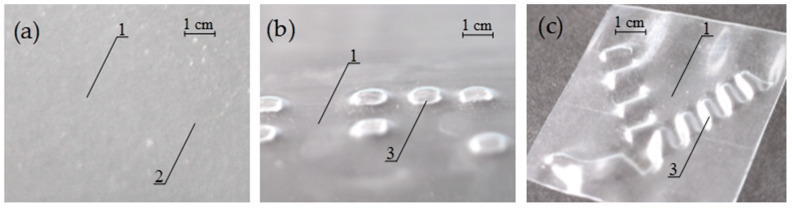
SMP PVC film after local heat treatment under isometric conditions (recording stage) and thermally stimulated free shrinkage (development stage). Tactile relief: (**a**) treated film before shrinking; (**b**) hot stamping; (**c**) laser processing. 1—shrink film, 2—hidden marking, 3—tactile (embossed) marking on the film.

Obtaining embossed characters and images on shrink film products, readable by a person visually and tactile, is based on the technology of interval materials [45,59]. The production of interval materials can be achieved by local heat treatment of a transparent SMP under isometric conditions using equipment for hot stamping [60], and by irradiation of an opaque SMP with laser light [61]. Interval materials have no relief and, as a rule, do not differ from the initial films in color, shape, and type of surface. Subsequent heat shrinkage of the interval materials in gaseous or liquid coolants shows the intervals subjected to thermal action in the form of local bulges of a given height and shape [44]. The height and shape of the relief depending on the modes of two-stage heat treatment of the film during marking are shown in Figure 5.

**Figure 6:**
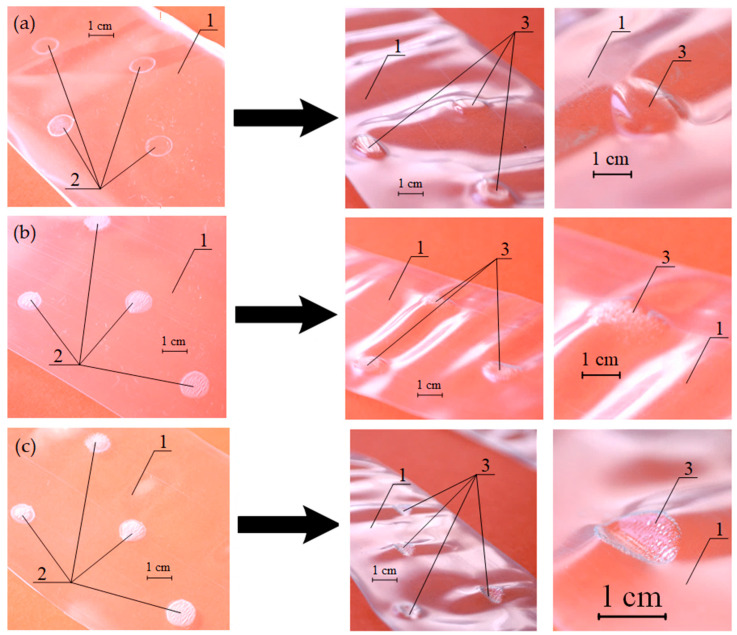
PVC films with hidden reliefs before and after heat treatment in a 30% THF solution at (**a**) 22 ± 1 °C; (**b**) 36 ± 1 °C; (**c**) 65 ± 1 °C. 1—shrink wrap, 2—hidden marking, 3—tactile (embossed) markings.

According to the recently proposed and patented method [58], interval materials and subsequent relief marking of shells from heat-shrink films can be obtained by partially dissolving and/or local swelling of the polymer in a liquid [46], printing varnish, or ink containing an active solvent of the polymer forming the heat-shrink film. In this case, a good solvent should have a thermodynamic affinity for the polymer and high volatility (low boiling point). After applying a liquid containing a solvent that quickly penetrates the polymer film by diffusion, internal stresses decrease in the heat-shrink film, the chemical composition of the ingredients, the supramolecular structure, and, as a consequence, the relaxation rate during subsequent shrinkage on the marked object change [44]. The height and shape of the information relief depend on the modes of two-stage heat treatment of the film during marking (Figure 6).

The height of the convex relief elements in Figure 6 depends on the level of internal compressive stress relaxation in the areas of the film exposed to the solvent and temperature. The intensity of this effect is determined by the thermodynamic affinity and concentration of the solvent, contact time, and temperature. The influence of these factors was evaluated experimentally by measuring the absorption of the THF film from an aqueous solution. The expected acceleration of absorption with the increase of temperature and concentration of the aqueous solutions is “masked” by dissolving the polymer or washing the ingredients out of the film. The composition of the PVC film includes liquid ingredients: dioctyl phthalate, polyphenylmethylsiloxane, and epoxidized soybean oil. The processes of polymer dissolution and absorption of THF, which compete in changing the film mass, reduce the level and rate of internal stress relaxation, and destroy the crystalline structures of the heat-shrinkable polymer film that hold the macromolecules in an oriented stress-strain state.

**Figure 7:**
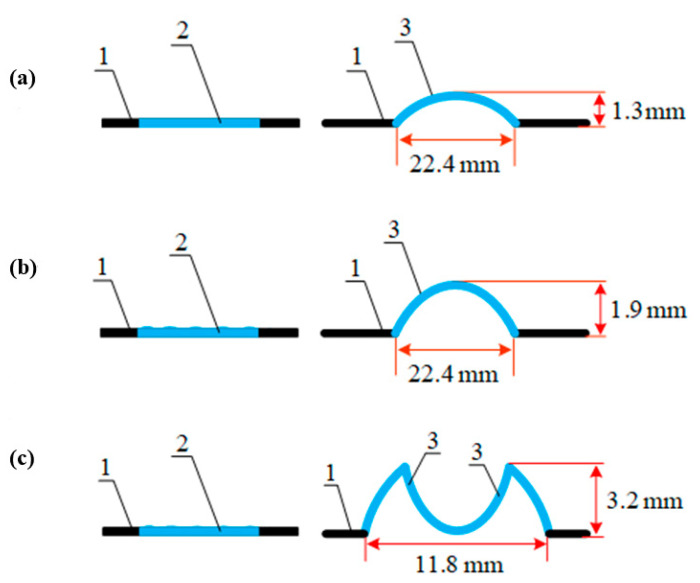
Section of a label made of an SMP film treated with a 30% THF solution before and after heat shrinkage in the applicator’s heat chamber [44] at (**a**) 22 ± 1 °C; (**b**) 36 ± 1 °C; (**c**) 65 ± 1 °C. 1—shrink film, 2—hidden marking, 3—haptic (embossed) markings.

According to the results of measuring the film mass after one-sided contact with the solution (Table 1), the conditions for the implementation of the marking method are determined, providing the height and width of the relief necessary for each specific object and the purpose of its marking. This can be seen in Figure 7 for marking labels or casings on objects.

It was found that the change in the mass of the PVC film caused by increasing temperature and processing time is not monotonic (Table 1). This is because the process of absorption of THF by the film competes with the processes of washing out low molecular weight ingredients of the polymer composition and dissolving PVC. The kinetic curve of the dependence of weight on time of the PVC film immersed in THF has a maximum. The extremum coordinate on the time scale depends on the solution concentration and temperature.

At a concentration of an aqueous THF solution of 30%, the increase in the film weight due to the absorption of organic matter from the solution reaches 19.2% in 14 s. A longer contact of the film with a THF solution or an increase in temperature up to the glass transition temperature of polyvinyl chloride (65 °C) does not lead to an increase in the mass of the samples due to the extraction of low molecular weight components of the polymer composition forming the film. At a concentration of an aqueous solution of THF of 40% and 50%, a similar effect of temperature on an increase in the mass of the film is observed. The temperature of maximum absorption is 36 °C, and the time to reach the maximum increase in the film weight decreases from 14 to 10 s. This time interval is comparable with the technically possible maximum contact time of the printed material and the stencil form of modern printing equipment [52].

The time to reach the maximum mass of the PVC film as a result of sorption of THF from an aqueous solution, provided that the solution contacts one surface of the film, is proposed as a measure of the effectiveness of modifying the structure of the film. This time is a criterion for choosing a method, type of equipment, and printing speed on SMP films.

**Figure 8:**
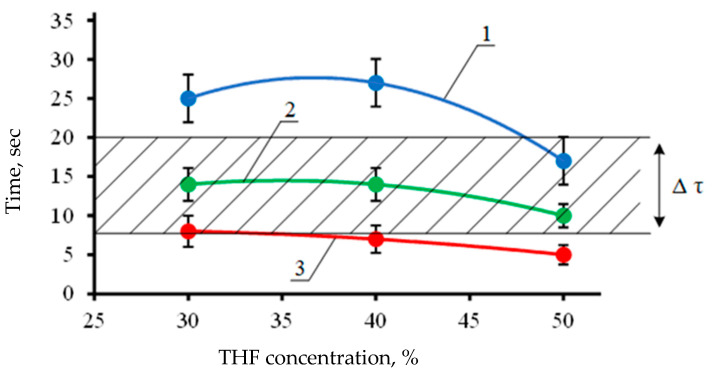
Swelling time to maximum absorption versus the THF solution concentration. 1–22 ± 1 °C, 2–36 ± 1 °C, 3–65 ± 1 °C. Δτ is the time interval of contact between the liquid and the film.

Using a roll-to-roll (R2R) setup, based on the printing equipment design and throughput, the liquid-to-film contact time can vary widely from 1 to 40 s. This time is calculated from the moment the ink droplets are applied to the film until it is wound into a roll. When using equipment for screen printing (such as “Gallus 340” [60]) with a cylindrical mesh and an extended drying section, the contact time can range from 7 to 20 s. To record information on SMP films, it is necessary to experimentally determine the critical temperature and concentration of the THF solution. The effects of THF solution temperature and concentration on the maximum saturation time of the PVC film are shown in Figure 8. The optimal condition for recording information on a tape with shape memory in tactile form using commercial printing equipment is 30% THF concentration at 35 ± 5 °C.

Figure 8 also shows the time interval Δτ corresponding to the technologically determined optimal time of contact of the film with liquid on various industrial printing machines without significant modification of printing assemblies.

## 4. Discussion

**Figure 9:**
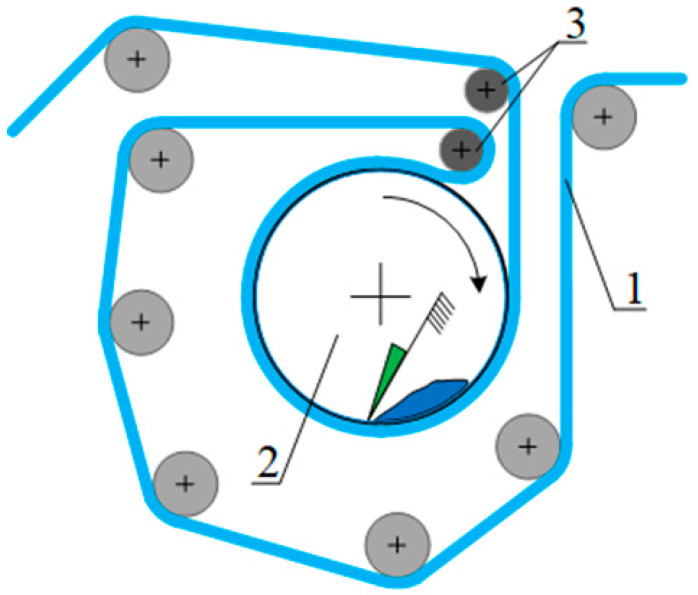
Diagram of a section of a screen printing machine with additional rollers pressing the film to the surface of a rotary printing plate with an increased diameter. 1—shrink film (shell) with shape memory, 2—printed form (metal mesh), 3—pushing rolls.

To apply the THF aqueous solution to the surface of PVC shrink film, it is proposed to upgrade the printing equipment. Screen printing equipment modifications need to increase the contact time of the PVC shrink film with the THF-containing paint and optimize the solvent absorption process temperature by the polymer to accelerate internal stress relaxation [44]. The increase in contact time can be achieved by two ways of upgrading the printing units. The first option is to manufacture a cylindrical printing plate with a large diameter, and thus increasing the maximum possible film coverage, as shown in Figure 9. The second option is to increase the number of rollers and the distance between them before the film is transported to the drying chamber under tension to prevent solvent-induced warping and shrinkage.

**Figure 10:**
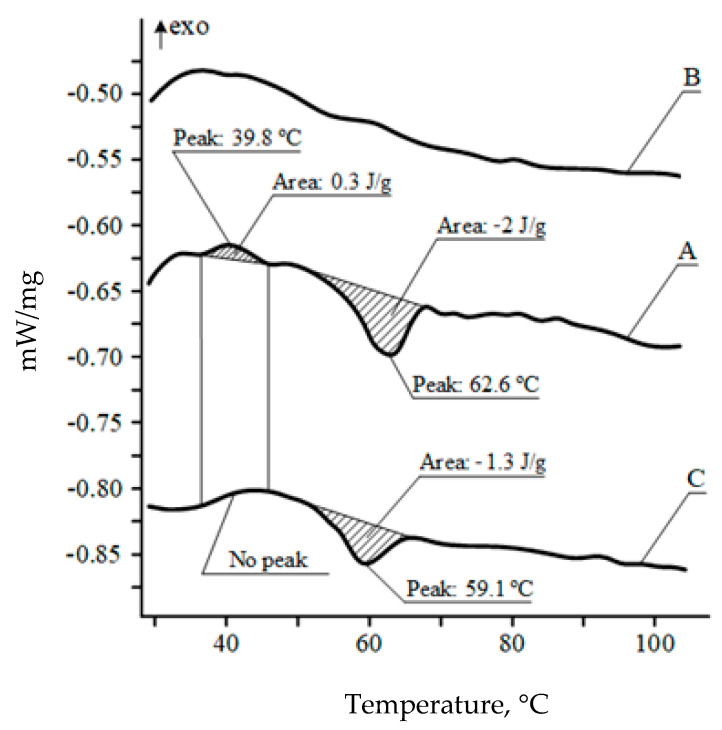
DSC analysis of PVC films with “shape memory”: A—”initial” film sample (1st heating), B—”initial” film sample (2nd heating), C—film sample treated at 36 ± 1 °C with 30% THF solution for 10 min.

Film coverage of a rotary screen printing plate is currently not used in traditional paper and film printing processes [60]. Increasing the contact time of the heat-shrinkable PVC film with the THF solvent can be carried out on gravure or screen printing machines by passing the film through a heated table or an extended drying conveyor, where, while removing excess solvent from the film surface, the diffusion of THF into the polymer continues and the stress relaxation process seals the printed areas. Changes to the internal stresses in a treated film using a modified R2R screen printing section (Figure 9), or the described laboratory technique, are confirmed by the DSC results in Figure 10.

The effects of solvents on the SMP structure differ from the effects of heat [60] or laser radiation [61]. The solvent initiates two simultaneous processes changing the structure of strained shape memory polymer film. When the solvent penetrates the polymer, the internal stresses accumulated in the film relax, which is reflected by the absence of an exothermic peak at 39.8 °C in Figure 10, curve “C”. The liquid also plasticizes the polymer and separates the crystalline formations, while reducing their size, which lowers the average melting point by 3 ± 1 °C and reduces the melting enthalpy by 30% [62,63].

## 5. Conclusions

Structural and phase transformations in an SMP film treated with an aqueous solution of THF allow using modernized printing equipment for recording information in the form of relief. The recording process is performed in two stages. The first stage is the formation of film materials with inhomogeneity of internal stresses using solvents, confirmed by DSC analysis. In this stage, hidden information, not observable visually or tactually, can persist for a long time. The second stage is the revealing of the information in the form of reliefs, which is carried out in the thermal chambers of industrial shrink label applicators used in the production and labeling of containers for packaging liquid and dry products.

The new technology for recording information on SMP film in the form of relief is designed to protect goods of famous brands, drugs, and hazardous household chemicals from falsification and facilitate the identification of goods by people with impaired vision. An important advantage of the proposed technology in comparison with known methods of embossed marking of polymer products (3D printing, thermoforming, stamping) is the ability to apply hidden protective symbols and brand indicators on special packaging details: brands, labels, complex labels, which are not organoleptically determined without additional heat treatment. Embossed symbols appear only in the process of verifying the authenticity of the package by shrinking such elements in a stream of hot air or water. Development of the design of protective packaging elements for specific goods and methods of verifying its authenticity is a separate task carried by manufacturers of brands especially valuable or dangerous products subject to protection from counterfeiting. The combined use of printed identification codes and embossed Braille inscriptions on SMP film packaging significantly increases the level of protection of goods from counterfeiting. Continued development of technology for recording, storage, and reproduction of information on polyvinyl chloride films using shape memory effects will include research on the durability of preservation of hidden information, expanding the range of polymeric materials for the production of SMP film and solvents suitable for recording and evaluation of their hygienic properties. It is also necessary to assess the resistance of the relief to mechanical stress, pressure and abrasion, resistance to weather factors and possible temperature changes during storage and transportation.

## Figures and Tables

**Table 1:** Change in the mass of PVC film after one-sided contact with an aqueous THF solution.

Parameters	THF Aqueous Solution Concentration, wt.%								
	30%			40%			50%		
Temperature, °C	22 ± 1	36 ± 1	65 ± 1	22 ± 1	36 ± 1	65 ± 1	22 ± 1	36 ± 1	65 ± 1
Max increase in film weight, %	6	19.2	15.1	15.7	23.1	21.6	22.6	31.4	28.3
Time to max weight gain, sec	25	14	8	27	14	7	17	10	5

## Data Availability

The data presented in this study are available on request from the corresponding authors.
